# Lockdown and No Lockdown: How Norwegian and Swedish Elite Athletes Managed Preparations for Tokyo 2020 and Mental Health Challenges in the Shadow of COVID-19

**DOI:** 10.3389/fspor.2022.918825

**Published:** 2022-08-02

**Authors:** Carolina Lundqvist, Elsa Kristiansen

**Affiliations:** ^1^Department of Behavioral Sciences and Learning, Linköping University, Linköping, Sweden; ^2^Athletics Research Center, Linköping University, Linköping, Sweden; ^3^Department of Business, Strategy and Political Science, USN School of Business, University of South-Eastern, Drammen, Norway

**Keywords:** mental disorders, Olympic Games, pandemic, resiliency, wellbeing

## Abstract

The present study explored Norwegian and Swedish Olympic aspirants' perceived challenges for the preparations of Tokyo 2020 Olympic Games (OG) and risk and protective factors for mental health. The focus for this study was the timespan between the declaration of the postponement of Tokyo 2020 and the final months before the Games. A secondary purpose was to explore experiences of both elite athletes affected by lockdown (i.e., Norwegian athletes) and elite athletes not affected by lockdown in their home country (i.e., Swedish athletes). Twelve elite athletes (Norwegian: *n* = 6; Swedish: *n* = 6; Women: *n* = 6; Men: *n* = 6) with a mean age of 28.25 (*SD* = 3.60) participated. Semi-structured interviews were conducted between April and June 2021. Seven athletes had qualified and five were still trying to qualify. Eight of the interviewed athletes had previous experiences with OG participation. Template analysis revealed two main themes: *(a)* challenges and risk-factors for mental health and *(b)* protective factors. The pandemic exposed athletes to several psychological strains like uncertainty and difficulties with planning and preparations for the OG and personal and social challenges (i.e., worry about physical health and risk of overtraining, social contacts, identity, and life issues). Protective factors included perceived benefits of increased recovery and time for quality training. The athletes used several coping strategies and self-care behaviors (e.g., focus on the controllable, playfulness, putting sports in perspective, daily routines, short-term goals, working or studying for personal development) and they tapped into various internal and external psychosocial resources perceived as protective for mental health, personal growth, resiliency, and adjustment to the pandemic. The holistic perspectives used contribute to an increased understanding of elite sport athletes' mental health needs in stressful and unforeseen situations such as a pandemic.

## Introduction

In December 2019 a new Coronavirus was discovered in China and on March 11, 2020, the World Health Organization declared a pandemic (World Health Organization, 2020). Countries all over the world went into lockdowns, and the pandemic had a direct impact on sports and competition at all levels (Evans et al., 2020; Samuel et al., 2020). One exception was Sweden where society, including training venues, was kept open for the complete pandemic period but with recommendations and rules from The Public Agency of Sweden for citizens to follow (Weman Josefsson, 2021). Three critical phases affected elite athletes during the COVID-19 pandemic and their preparations for Tokyo 2020, which included the phase before the postponement, during the postponement, and during the reactivation of elite sports for Tokyo 2020 (Schinke et al., 2020a). Elite athletes were, at the time of the pandemic outburst, trying to qualify or had already qualified for the Tokyo 2020 XXXII Olympic Games (OG). The declaration of a pandemic imposed great uncertainty around Tokyo 2020 (Schinke et al., 2020a; Taku and Arai, 2020; Lundqvist et al., 2022). On March 24, 2020, the International Olympic Committee (IOC) made the historical decision to postpone Tokyo 2020 to the summer of 2021 at the latest. The pandemic and postponement of the OG was a stressful situation for elite athletes challenging their mental health; athletes had to change their training routines, short and long-term goals and therefore lifestyles (Taku and Arai, 2020; Lundqvist et al., 2022; Stambulova et al., 2022).

There is a growing consensus among major sports organizations, including the IOC, that the protection of elite athletes' mental health is a priority (Reardon et al., 2019; Vella et al., 2021). The World Health Organization (2004) defines mental health as “a state of well-being in which the individual realizes his or her own abilities, can cope with the normal stresses of life, can work productively and fruitfully, and is able to make a contribution to his or her community” (p. 12). The pandemic increased sport organizational strains (e.g., changed OG selection criteria, planning, coach strategies), which are known to threaten athletes' mental health (Fletcher et al., 2006; Arnold and Fletcher, 2012; Arnold et al., 2017; Rice et al., 2021). In addition, restrictions and lockdowns challenged athletes' motivation and created a need to reinvent safe training and competition opportunities (Elliott et al., 2020; Löllgen et al., 2020; Lundqvist et al., 2022). The pandemic was anticipated by several researchers to have negative effects on elite athletes' mental health, for example, loss of meaningfulness and identity, loneliness, social isolation, stress, anxiety, and feelings of uncertainty about Olympic qualification, worry about finances and sponsorship, and sleeping problems (Schinke et al., 2020b; Toresdahl and Asif, 2020; Facer-Childs et al., 2021; Fröhlich et al., 2021; Haan et al., 2021; di Fronso et al., 2022; Stambulova et al., 2022).

Increased levels of stress and anxiety among elite athletes were reported by quantitative cross-sectional studies during the pandemic (Haan et al., 2021). Scholars reported, however, also functional adjustment to and benefits of the pandemic situation among elite athletes. For example, di Fronso et al. (2022) reported that elite/expert athletes, when compared to novice athletes, displayed lower self-rated stress levels and more functional psychobiological states. Clemente-Suárez et al. (2020) showed anxiety levels among Olympic and Paralympic athletes to be comparable to non-pathological populations, suggesting that the quarantine had not impacted their anxiety responses. Some studies also argued that elite athletes, despite a mixture of emotional reactions, were resilient and able to even see the benefits in the situation (e.g., mental and physical recovery, increased time for preparation for the OG, time for reflection on sports and life; Oblinger-Peters and Krenn, 2020; Lundqvist et al., 2022).

Although several risk-factors for mental health were present during the pandemic year when Tokyo 2020 was postponed and challenged athlete mental health, it is also plausible that athletes had access to various protective psychosocial resources to cope with the situation and their emotional reactions. For example, coach support, the coach-athlete relationship and social support are known to be essential for athletes to manage various stressors (Jowett and Cockerill, 2003; Kristiansen et al., 2012a). Social support can in addition be provided in various forms, often classified based on its content in terms of emotional, informative, or tangible support (Schaefer et al., 1981), defined as “social interactions aimed at inducing positive outcomes” (Bianco and Eklund, 2001, p. 85). Elite athletes are likely to adopt various emotional regulation strategies to manage perceived stress. Emotional regulation choices can vary depending on several determinants, for example affective factors (e.g., emotional valence, intensity), demographic factors (e.g., age, gender), individual traits (e.g., self-esteem, attitudes, cognitive resources), motivation (e.g., goals), and social-contextual determinants (e.g., need of belonging, norms) (Matthews et al., 2021).

Considering the complexity and multidimensionality of elite-sports environments, researchers increasingly advocate the use of a whole system perspective on the study of elite athletes' mental health (Purcell et al., 2019; International Olympic Committee, 2021). For example, Purcell et al. (2019) suggest a framework in which elite athletes' mental health and support efforts are considered within a broader ecological sports system, with interactions between the “microsystem” (e.g., coach(es), teammates, family), the exosytem (e.g., the sport the athlete is involved in), and the macrosystem (national and international sports federations, society). Similarity, Lundqvist (2021) suggests a holistic model where athletes' mental health is evaluated in the context of goals and values in life and sports, biopsychosocial factors, and culture. Mental health as a construct can in addition be regarded as an umbrella term that comprises everything from wellbeing to psychiatric diagnoses of various severities (Huppert, 2009; Keyes, 2014; Lundqvist and Andersson, 2021). Mental health can therefore refer to daily and transient moods or psychological reactions to life events as well as clinical conditions defined by the Diagnostic and Statistical Manual of Mental Disorders-5 (DSM-5; American Psychiatric Association, 2013) or the International Statistical Classification of Diseases and Related Health Problems-11 (ICD-11; cf. Pocai, 2019; World Health Organization, 2022). To add depth to the findings from quantitative cross-sectional studies on elite athletes' mental health during the pandemic, we chose a qualitative approach to understand elite athletes “from inside their subjective experiences” (Mayoh and Onwuegbuzie, 2015, p. 92). The present study explored Olympic aspirants' experiences with challenges to their preparations of Tokyo 2020 Olympic Games (OG) and perceived risk and protective factors for mental health. This study focuses on the timespan between the declaration of the postponement of Tokyo 2020 and the final months before the Games. Sweden and Norway are culturally similar countries, but Norway went into lockdown while Sweden did not. A secondary purpose was therefore to explore experiences of both elite athletes affected by the lockdown (i.e., Norwegian athletes) and elite athletes not affected by lockdown in their home country (i.e., Swedish athletes).

## Materials and Methods

### Participants and Procedure

All athletes were recruited by purposive sampling using the criteria: (a) the athlete had qualified for Tokyo 2020 or had a realistic opportunity to qualify, and (b) competed in an individual Olympic sport. To enable a variety of experiences to emerge, the elite athletes were also purposively sampled to obtain heterogeneity in terms of sports included. Twelve (men: *n* = 6; women: *n* = 6) individual elite athletes (Norwegian athletes *n* = 6; Swedish athletes *n* = 6) with a mean age of 28.25 (*SD* = 3.60) participated. The sports represented were athletics, cycling, table tennis, gymnastics, judo, rowing, sailing, swimming, and wrestling. As shown in Table 1 and based on the elite athlete definition proposed by Swann et al. (2015), four athletes (*n* = 4) were classified as world-class elite athlete (i.e., had repeatedly won medals at the highest sports level over many seasons), five athletes (*n* = 5) as successful-elite athletes (i.e., competing and winning at least one medal at the highest sports level), and three athletes (*n* = 3) as competitive-elite athletes (i.e., competing at highest sport level without winning a medal at that level). Eight of the participants had previous experiences of participating at the OG. At the time of the interviews, seven athletes (Norwegian: *n* = 3; Swedish: *n* = 4) had qualified for the OG and five were still trying.

**Table 1:** Descriptions of the 12 elite athletes' sports levels and merits.

**Participant no**.	**Nationality**	**Gender**	**Qualified for OG**	**Years at elite level**	**Podium at International Championships**	Classification elite athlete*
#1	Swedish	Male	Yes	10	Participation at EC, WC. Bronze medal WC	Successful-elite
#2	Swedish	Male	Yes	15	Participation at EC, WC, and OG. Gold medals EC and WC	World-class elite
#3	Swedish	Female	Yes	15	Participation at EC, WC, OG. Gold, silver, bronze medals EC, WC and OG	World-class elite
#4	Swedish	Female	Yes	13	Participation at EC, WC, and OG. Silver medals EC	Successful-elite
#5	Swedish	Male	No	12	Participation at EC and WC. Top five in EC and WC	Competitive-elite
#6	Swedish	Female	No	16	Participation at EC, WC, and OG. Bronze medal EC	Successful-elite
#7	Norwegian	Male	Yes	15	Participation at EC, WC, OG. Gold, silver, bronze medals EC, WC and OG	World-class elite
#8	Norwegian	Male	Yes	7	Participation at EC, WC and OG. Silver and bronze medals EC and WC	World-class elite
#9	Norwegian	Female	Yes	12	Participation at EC, WC, and OG. Bronze medal WC	Successful-elite
#10	Norwegian	Female	No	11	Participation at EC, WC. Top five in EC and World-Cup	Competitive-elite
#11	Norwegian	Female	No	9	Participation at EC, WC. Top five in EC and World-Cup	Competitive-elite
#12	Norwegian	Male	No	8	Participation at EC, WC. Gold medal EC	Successful-elite

*Classification based on Swann et al. (2015). EC, European Championships; WC, World Championships; OG, Olympic Games.

In accordance with Norwegian and Swedish national ethical standards, procedures were approved by the ethical board in each country before data collection. All athletes were asked to participate by email or by telephone and contact information was provided by federations, clubs, or similar contacts. Participants were given written information about the purpose and details of the study as well as information about ethical considerations. All contacted athletes agreed to participate and provided informed consent. The interviews took place between April and July 2021 via Zoom. The interview followed a semi-structured format with open questions and follow-up questions covering: (a) Demographics, athletic background, experiences from championships, short and long term goals, overall situation during the pandemic situation (e.g., dual-career, work), and (b) Questions about the everyday sports context from March 2020 until the present, including challenges and benefits for sports and mental health as well as risk and protective factors for mental health, coping strategies, and social support. All interviews were recorded and transcribed verbatim. The interviews lasted between 40 and 90 min. Both interviewers were familiar with the elite-sport context, and they have been close to the elite sport context for more than a decade. The first author is a sport psychology researcher, sport psychology consultant and licensed psychotherapist, working with elite athletes, including Olympic athletes. The second author is a sport psychology researcher who has done several research projects on high-level elite sports.

### Data Analysis

Thematic analysis in the form of template analysis was adopted (for an overview see Brooks et al., 2015) underpinned by a pragmatic epistemology and combining an inductive and deductive approach (Roberts et al., 2019). Template analysis is a flexible approach where a subset of data initially is used to create a first hierarchical coding structure and initial theme definitions (Brooks et al., 2015). The steps for conducting template analysis described by King (2012) were followed whereby the authors started with reading the data in full. Secondly, a first preliminary coding template was developed deductively to keep a relatively high degree of structure in the analysis. Initial themes in the first coding template used included athletic challenges and protective factors for athlete a mental health. Both authors individually coded two transcripts of interviews based on the two themes a priori identified. Subcategories that emerged in this initial phase were thereafter critically discussed between the authors and used to further develop the coding template. The analysis thereafter continued where both authors kept a high flexibility to the data to inductively uncover new perspectives and themes that emerged. The coding template was continuously discussed and developed by the authors and adjustments were performed based on the progress of the analyses. Additional themes were added to the coding template when appearing in the data. In the final phase of the analysis, themes found being closely related were combined into broader themes and organized hierarchically into main themes and sub-categories. In studies using template analyses it is common that themes can have several levels of sub-categories (Brooks et al., 2015) and in this study it was decided that two to three levels of sub-categories covered the content of data. Throughout the research process recommendations by Smith and McGannon (2018) were followed to develop rigor. Throughout the process of data-collection, analysis, and presentation of the results, the two researchers repeatedly discussed interpretations as critical friends. Member reflections were used with some participants to create high quality and meticulous research and to generate additional insight into the process. Additionally, results were discussed with colleagues (e.g., researchers, practitioners) to obtain critical feedback and improve organization of themes and encourage reflection on interpretations of the results.

## Results

The results are presented based on two main themes and are also summarized in Tables 2, 3: (a) challenges and risk-factors for mental health and (b) protective factors. We present the Norwegian and Swedish experiences together, pointing at major differences. To protect the participants' anonymity, sports and other identifying details are removed.

**Table 2:** First main theme: athletic challenges and risk-factors for mental health.

**Second level categories**	**Third level categories**	**Examples mentioned in the interviews**
Uncertainty and planning related to training and OG preparations		• Training without breaking restrictions • Lack of sparring • Olympics or not? • Changed plans, rescheduled/canceled training camps and competitions • Difficulties evaluating training progress • Travels time-consuming and stressful (PCR-tests, quarantine rules) • Few competitions • Economical stress
Personal and social challenges	Physical health and risk of overtraining	• Worry getting sick and not being able to practice • Training planning: Training to much without breaks
	Social contacts	• Less contact with friends within and outside sports • External critique about participating in the OG during a pandemic
	Identity and life issues	• Questioning who I am • Rescheduling life • Last chance in the career to participate in OG • Life to narrowly focused on OG • Losing sight of long-term goals

**Table 3:** Second main theme: protective factors.

**Second level categories**	**Examples mentioned in the interviews**
Benefits of recovery and training	• Recovery of injuries without stress • Less stressful life and relaxation at home • Time for quality training to reach a higher athletic level
Specific coping strategies and self-care behaviors	• Focus on sports development, your performance and what is controllable • Remembering why you do sports and taking ownership of actions • The importance of playfulness, down-play being “the best” all the time • Acceptance and psychological adjustment to the pandemic situation • Routines, short term goals, variation and positive reinforcement in training and life
Long-term personal growth and resiliency	• Personal development and broadened competencies outside sports by studies and work • New experiences and altered view on training and need of recovery • Appreciation of what you take for granted • Perspectives on elite sports • Experience that you can manage it even if a situation is chaotic in the moment
Social support	• Availability of social support and a sense of coherence

### First Main Theme: Challenges and Risk-Factors for Mental Health

The first main theme was challenges and risk-factors for mental health which included two sub-categories: (a) uncertainty and difficulties with planning, and (b) personal and social challenges.

#### Uncertainty and Difficulties With Planning

The greatest challenges expressed by the athletes had to do with the uncertainty and difficulties in planning imposed by the pandemic situation. This included general training logistics and OG preparations. Lockdown and its consequences for training logistics were major challenges for both qualified and non-qualified athletes in Norway because of the uncertainty and hardship in planning that all athletes experienced. The rules for what you were allowed to do were constantly changing and all interviewees expressed this as a challenge, exemplified by the following quote from a Norwegian athlete:

I like to plan, preferably six months ahead when it comes to travels … but with COVID-19 that was not possible, and I had to learn how to change my plans quickly. But I adjusted, and I do not spend time being depressed about what I cannot change or what is optimal.

Sweden did not go into lockdown, which made life easier for Swedish athletes. The Swedish athletes acknowledged that they, in comparison to their international competitors, were fortunate to have been able to train almost as usual although with some general restrictions: “It was possible to train as usual all the time, while in other countries it was much tougher” (Swedish athlete). Another logistically challenge was, as that only a few athletes in the two countries were at the highest level of sparring, inviting international athletes was necessary for training. Athletes mentioned that it was a challenge in sports with weight classes as they may only have “one in each weight class at the top” (Norwegian athlete).

Both Norwegian and Swedish athletes experienced advantages of qualifying for the OG before the pandemic outburst. One Norwegian athlete was especially clear on that: “I have been lucky to have qualified, so my whole focus has been to train as best I can so that I can be the best I can in Tokyo”. Uncertainty whether the OG would happen and around how to prepare for the OG during the pandemic was prevalent in the interviews with athletes from both countries. Another Swedish athlete expressed: “I think the most difficult thing has been the uncertainty and the adjustment that it was another year. Postponing the Olympics was a big deal”. The preparations for both Norwegian and Swedish athletes were challenged by the pandemic rules and frequent changes in restrictions, which meant they had to change plans and reschedule or cancel training camps and competitions. Some athletes also expressed difficulties with evaluating training progress in the absence of competitions. They found it difficult to evaluate whether they were heading in the right direction in their preparations, exemplified with this quote from one Swedish athlete: “getting feedback from competition and in other contexts. It has been important to have courage, to trust your own process”. Moreover, the second wave of the pandemic appearing in the autumn 2020 was experienced as challenging for motivation when athletes were in great shape, had started competing again, and then competitions were canceled again.

A common stressor for all athletes was the frequent PCR-testing and quarantine rules, which made traveling time consuming. As one Norwegian athlete put it:

I know that I am lucky that I can travel a lot and compete in some competitions abroad. But that all the time there are different rules, that there are different tests and when you need a test that you always… It becomes an extra burden all the time. That's probably what I think is the most difficult part.

In addition, Norway had entry rules with home quarantine after travels, resulting in training breaks for several weeks for the Norwegian athletes^1^. All Norwegian athletes who were in the OG qualification process agreed that these rules made it difficult to optimize training and compete. It negatively affected their opportunities to reach the OG, and it became an extra burden when they were infected with COVID-19 abroad:

We had the strictest entry rules in all of Europe, and thus we decided to travel for two longer trips. First a six-week training camp, at home for 2 weeks, and then for another 3 weeks... We took a risk to be able to fight for an Olympic ticket. It was tough qualifying. But before the competition, some of us got Covid, so then we were left abroad 2–3 extra weeks in. It was not optimal.

One Norwegian athlete who had sponsors explained the economic implications of not being able to compete and labeled it “the biggest challenge”:

One cannot expect sponsors to maintain athletic sponsorships when they must let people go because of the pandemic. Thankfully, I have solid sponsors who managed it well and made money, but there has been a decline in income. My federation only pays for training and competition expenses, the rest we need to pay ourselves.

#### Personal and Social Challenges

None of the elite athletes interviewed had suffered severe mental health problems during the pandemic period, however, several plausible personal and social risk-factors for mental health were mentioned by athletes. Personal and social challenges expressed by athletes included worry about physical health and risk of overtraining, social contacts and identity or life issues.

##### Physical Health and Risk of Overtraining

Personal and social challenges reported by athletes included worries about physical health and consequences for training. Worry about physical health was related to worry about getting infected by COVID-19 but also about other symptoms. Even mild symptoms could hinder them from practice: “Even if I get a little cold, I will not be able to train” (Swedish athlete). Some athletes also mentioned the risk of overtraining because normal breaks for competitions when athletes usually got some periodization with decreased training were absent:

You may train a little bit too hard or a little bit too long. It can be a risk. When you are about to compete, you decrease training a bit beforehand. Then you recover for a day after the competition. But if you just go on there will be very long training periods and then you become physically tired. And then it will eventually break you down mentally as well (Swedish athlete).

##### Social Contacts

Another challenge related to the decreased social contacts with friends caused by social distance restrictions and the absence of competitions:

A challenge for me is that I don't have as much social contacts as I used to have. The social contacts that you have with your international sporting buddies when you train and compete. I think this has been tough (Swedish athlete).

The same sentiment was also expressed by the Norwegian athletes and number of family members on spent time with became limited. Instead, the athletes spent much more time together their so-called “bubbles” (i.e., isolated sets of accommodations and venues in which athletes can reside and compete in small cohorts and without spectators). Another Swedish athlete mentioned that external critique about participation in Tokyo 2020 during the pandemic could be a risk-factor for mental health: “There could be hate from people who do not think it is okay to prioritize the Olympics when there are major problems in the world”.

##### Identity and Life Issues

Several athletes explained that they questioned their identity when not doing their sports. Some also emphasized the need for creating new daily routines when they could not train as usual and had more free time than they were used to. Moreover, athletes described that they also had time to reflect on the need to consider sports-life balance, including family life:

The change. We talked so much about how the months would be until the Olympics. Then suddenly, it [the Olympics] is postponed for another year. /…/. Life and sports are very close to each other. It's not the job and then life, they are highly interrelated. Sometimes the sport can jump into the private and vice versa. /…/ So, it has been a mix. /…/. It's not just you, you are not there alone on the podium, it's not just me. It is also loved ones and the sacrifices they make (Swedish athlete).

Knowing that this might be the last shot for an Olympic participation, or that the cancellation might lead to an early retirement could be stressful. Some athletes described that losing sight of long-term goals or a narrow elite sports identity could be a risk for elite athletes' mental health:

If everything is hung up very much on the Olympics. If it becomes your raison d'être for doing your sport, then I think it can be very difficult. If you, in combination with the choice to narrow your life and do a lot just for that... I think it can be a cocktail for mental illness (Swedish athlete).

Some older athletes had planned to retire as elite athletes after the Olympics and start a new life in the fall of 2020. For them, the postponement of the Olympics not only caused an extra year of preparations but also delayed their new life plans. Thus, these athletes had to re-plan life after the Olympics once again:

Usually, you have a plan and you know how to do it. If it cracks, then it's like you are standing there stomping. If you have a goal and, when I have reached it, I will do this for a while and then focus on the next goal. /…/. But it certainly depends on how old you are (Swedish athlete).

### Second Main Theme: Protective Factors

The second main theme shown in Table 3 were protective factors for the athletes' OG preparations and mental health during the year between March 2020 and the final months prior Tokyo 2020. Benefits of the postponement of the OG, coping and self-care strategies for managing challenges caused by the pandemic, personal growth and resiliency as well as social support emerged as sub-categories.

#### Benefits of Recovery and Training

Some athletes expressed that the OG postponement resulted in athletic benefits in terms of the possibility of recovery. Recovery could relate to injuries from which they could heal without the stress of an upcoming Olympics:

In March last year, I had just started training after being injured. I was in bad shape and felt stressed with a lot of alternative training. It was a nightmare scenario because the Olympics were coming, and I was still injured. Then it was postponed. It was not a relief, but it was more like I could calm down a bit. /…/ If you compare this with a year ago, I probably would not have been able to get there [to the Olympics] (Swedish athlete).

Mental recovery was also emphasized as a positive outcome by several athletes. Life became less stressful without travels and competitions, which meant they could spend more time at home perceived as relaxing:

Because we do much traveling, it's nice to be at home too. /…/. I would like to say that it is negative not to be allowed to travel and do my sport, but it is tiring to be away. To live two lives (Swedish athlete).

The COVID-19 pandemic also forced athletes' creativity on how to train, and some athletes chose to focus on advantages of getting more time for quality training to reach a higher athletic level without breaks for travels or competition:

There was endless time to train, which you normally do not have during a normal season with a lot of matches and travels. I felt it was really a chance that I took advantage of. Not everyone gets it, or it is not always you get that chance (Swedish athlete).

The Swedish athletes expressed that it was protecting for their mental health that Sweden did not go into lockdown and that training could continue the entire time.

#### Specific Coping Strategies and Self-Care Behaviors

All athletes talked about internal strategies and self-care to protect their mental health and stay motivated. They focused on their sports performance, development, and the controllable were, exemplified by this quote “…just try to focus on what you can focus on, it is on training and getting in good shape and then the rest will work out” (Swedish athlete). Remembering why you do sports was stressed by a Norwegian athlete:

I think it's important to remember why you do sports... and realize that not everything should be fun. … The reason that I do sport is to test my own limits, I like to come home and know that I have done a good job. If you only love competitions, or only love the attention, then you will not last.

Another Norwegian athlete stressed the use of mental skills and taking ownership of training and actions:

I have benefited greatly from the mental skills I have learned a few years back to take ownership of training and what I do. Knowing 100% why you do the training you do, and wanting to do it yourself, not because someone asks you to do it, you know you must do it to achieve the goals and to get better … This mental skill [owning it] helped me through it, and I have become very aware of the benefits of alternative training.

Moreover, several athletes stressed the importance of downplaying the need to be “the best” all the time and to remember playfulness despite being an elite athlete:

But also do things that you think are fun. Those things that made you start with sports from the very beginning. Go back to playfulness. It does not matter if you are 25 or 15 years old. I think many [elite athletes] lose their playfulness (Swedish athlete).

Other strategies included acceptance of and adjustment to the pandemic situation, keeping or finding new structures and routines, finding positive things in everyday life, and focus on short-term goals. One Swedish athlete used the metaphor of a “marathon race” to exemplify strategies used during the pandemic year:

Split the marathon race into different sections. Then I think it's important to find the small things that stand out in everyday life /…/. I really think you should mix things up a little. Previously it could be when you had a competition. When you don't get to travel, then it's important to be creative. We all have different things that give us energy, things that give life a little bit color.

One athlete mentioned patience as an advantage and that things had never come easy to him. In contrast, one Norwegian athlete was so disappointed when the Olympics were canceled that he took a long break. This was a self-care strategy and something this athlete would not have done under normal circumstances.

When COVID-19 hit us and the Olympic was canceled, I took a break from March 2020 until July. Then I started training again and preparing for the European Championship in the fall.

#### Long-Term Personal Growth and Resiliency

Several athletes expressed they had used the time for personal development and had focused on broadening their competencies outside sports, for example by going back to school. Being able to do so, made one express: “It also made me feel that I have developed all the time, even though it's been a bit stagnant in sports. I have moved forward in life on another level” (Swedish athlete). In the interviews, athletes emphasized personal growth and gaining new perspectives when asked about any learnings during the pandemic. To put things and sports in perspective was also a strategy used by several athletes: “To see what is most important. Sports are my profession right now, that is what I do and what I spend my time on. But it is also just sports” (Swedish athlete). Athletes also mentioned that the situation had taught them that they could train differently and that they appreciate recovery.

The pandemic also forced athletes to rethink their life situation, and when not being able to compete – they realized how much they loved their sport. One athlete explained that the pandemic probably had helped to prolong the elite sports career with a few years:

You should not take travel and competition for granted. There will come a time when you may not be an elite athlete anymore. You have experienced what it is like not being able to have these competitions, not being able to go to them (Swedish athlete).

An overall perception of increased resiliency was expressed in several interviews; athletes learned that they could excel even when faced with extreme challenges, exemplified by this quote from a Swedish athlete: “I can handle a pandemic without losing motivation”. One Norwegian athlete expressed the importance of optimism:

I am a realistic optimist; I have never let doubt take any place in my head. I always thought that the Olympics would be held in 2021, even though there was probably a 70% chance, but I just had to think like that, and then you just prepare for it. I have done what it takes to be ready for the Olympics.

#### Social Support

Social support, a sense of coherence and appreciating one's team-mates was also expressed as protective for mental health. Many athletes highlighted the importance of the team in this situation: “I cannot imagine what it is like to play sports without a team...it is also nice to belong to a national team” (Norwegian athlete). Support from the coaches, support staff, federations, and other athletes was greatly stressed as well as relationships with family and friends. Some coaches arranged digital team meetings when restrictions made face-to-face meetings impossible:

What my international club did well, when the pandemic started, was that we had a Zoom meeting with everyone. Everyone in the group. Even though we were in different countries and some in Africa, some in England, some in Sweden. It was nice to just talk and share how the restrictions were in all countries (Swedish athlete).

One Norwegian athlete pointed out that this period gave them time to work on group processes within the team, which in addition made the group cohesion stronger:

At the same time as COVID-19 hit us, some at our team used the pandemic as a pretext to start a process to change how we train. We found ourselves in an awkward position, it was some sort of chaos until the coach gave an ultimatum to stay on the team … The coach took control, training was coordinated much better, and we worked on group processes and had meetings on Zoom every week. It was good. When we finally could leave for camp, everyone was on the same page.

Finally, some Norwegian female athletes mentioned in a subclause that it may be easier to get support and have family around you as a male elite athlete. This was related to all types of social support including economic security, priority when it comes to planning and resources and at the personal level:

It is hard to live alone during a pandemic, and I think it is easier to be a man elite athlete than a woman, because women will more easily prioritize boyfriends and other people's wishes. It is not so easy to find a man who is willing to give up everything to support you and your dream.

## General Discussion

The COVID-19 pandemic and the postponement of Tokyo 2020 added to the stressors elite athletes normally encounter when preparing for an OG. The main challenge expressed by both Norwegian and Swedish athletes was the tremendous uncertainty caused by rapid shifts during the pandemic's different waves and the subsequent effects on conditions, posing difficulties in planning. All the athletes perceived the restrictions to be challenging, and the Norwegians more than the Swedish due to the number of regulations to follow. Mental health is vital for sustainable Olympic sports participation and success. This qualitative study adds to the knowledge base we have from previous cross-sectional and quantitative studies by providing athletes' unique perspectives on OG preparations and mental health during the complete COVID-19 period.

Notably, the pandemic and the postponement of the OG were not automatically experienced as unilaterally negative by all athletes. Or, alternatively, athletes interviewed in this study were skilled in problem-focused coping (Lazarus and Folkman, 1984) and they were able to reframe challenges in a more positive light after years of practice. In similarity to results found by Oblinger-Peters and Krenn (2020), several athletes reported benefits, for example, opportunity for mental recovery due to less stress, limited travels, and increased team cohesion. Some took the opportunity to heal from injuries or having an extra year for athletic development and training. This opportunity enabled them to be better prepared to qualify to Tokyo 2020, which may not have been possible if the Games had been held when originally planned. An encouraging finding in the present results is that athletes use several successful coping strategies and self-care behaviors and that they tap into various internal and external psychosocial resources for support. Elite athletes are frequently exposed to adversities, uncertainty, and stressful situations in their elite sports lives, which makes it necessary to develop functional coping skills and emotion regulation strategies. It is plausible that these skills and strategies were transferred to cope with the pandemic (Clemente-Suárez et al., 2020; Oblinger-Peters and Krenn, 2020; di Fronso et al., 2022). In the current study it was found that athletes described acceptance of the pandemic situation, and the fact that Sweden did not go into lockdown, which made it possible to train the entire pandemic period, as protective for their mental health. Other scholars have reported similar results. Maintaining physical activity, the availability of sports equipment and possibility to continue with training were, for example, reported as protective for Spanish elite athletes' mental health during lockdown (Jaenes Sánchez et al., 2021; Moscoso-Sánchez et al., 2021). Jaenes Sánchez et al. (2021) also found that acceptance of the need of social isolation during the pandemic was related to positive feelings like friendship. Moreover, both in the current study and in previous research (e.g., Abenza-Cano et al., 2020; Izzicupo et al., 2021), dual careers are emphasized as an important protective factor for elite athletes. Participants in this study described that turning to work or studies helped them grow as persons outside sports when development in sports was challenged.

Leaning on social support was a common theme among the athletes. Support described by the athletes was informational (i.e., support in form of feedback on development from coaches and information about training camps and championship from federation or the National Olympic Committee), tangible (i.e., support in form of direct aid through funding, sponsor support and logistics) and emotional (i.e., support from friends, family, and coach/support personnel; Schaefer et al., 1981). The interviews underlined that warm, heartfelt coach-support is pivotal for coping, a finding that has been reported previously (Jowett and Cockerill, 2003; Kristiansen and Roberts, 2010; Kristiansen et al., 2012a). Coach support was for some provided by a national coach, while for others a local or international club coach was closer to the athlete, depending on where they were situated and trained. Some female athletes argued that their male counterparts seemed to be more supported. Staff and organizations who are in a position to support athletes' wellbeing should keep in mind that making extra support available in unpredictable times is important for all but may be expressed differently across gender, sports samples, and cultures (Kristiansen et al., 2012b). While Scandinavian countries are often mentioned to be at the forefront of equality, men continue to win 75 % of Norway's Olympic medals (Haugli, 2018). There are of course several reason for this, though structural changes and more support might be needed to improve the women's statistics (Kristiansen and Berntsen, 2021). A pandemic made the differences even more notably, and these findings are supported by a recent study conducted by Bowes et al. (2020). The interviewed elite women athletes perceived they had less access to equipment and that men's sport was prioritized. Also, the financially side of sport and the impact through a reduction in match fees or sponsorship, or furlough were argued to hit the women harder (Bowes et al., 2020).

Many sport-specific risk-factors for athlete mental health are modifiable at different levels in the sporting context, and as shown in the current study, risk- and protective factors commonly involve the individual athlete, the close context around the athlete (i.e., coaches, family, teammates) as well as the context within the wider sports environment (see also Purcell et al., 2019; Reardon et al., 2019). The findings in this study emphasize contentions by other scholars who have stressed the importance to consider various levels of the ecological sporting context to enable a comprehensive understanding of both risk and protective factors and their interaction in relation to athlete mental health (Purcell et al., 2019; Kuettel and Larsen, 2020; Lundqvist, 2021; Simpson et al., 2021). Empirical knowledge of protective factors among sports populations has, however, until recently received sparse attention in sports psychology research and still constitute a limiting factor for the development of support systems targeting both performance and mental health among elite athletes (Breslin et al., 2017; Kuettel and Larsen, 2020). Sport-specific and evidence-based mental health promotion approaches that can be applied by practitioners to support athlete wellbeing, especially in times of crisis such as pandemics, has also been limited because of few well-designed studies establishing the efficacy (i.e., randomized controlled trials) and effectiveness (i.e., pragmatic trials) in sports settings (Breslin et al., 2017; Lundqvist et al., 2022).

Several psychotherapeutic approaches and treatments are available in the clinical psychology, which likely are valuable also to support common mental health concerns in athletes when life or sports burdens exceed the athlete's resources to cope with them (e.g., Lundqvist, 2020; Barlow, 2021; Reardon et al., 2021; American Psychological Association Division 12, 2022). For example, exceeded and prolonged uncertainty or stressful life changes, as was found among athletes in this study, is associated with increased levels of anxiety or depression among athletes (e.g., Gouttebarge et al., 2019). Low-intensity CBT is a brief and evidence-based intervention with a “here and now” and skills-based approach that has demonstrated efficacy and effectiveness for several psychological concerns including worry and depression (e.g., Bennett-Levy et al., 2010). Low-intensity CBT-interventions could, if used as an approach by support staff with adequate training, be an option when preventively supporting elite athletes to increase their coping ability and resiliency in stressful and uncertain situations like pandemics. Communication between key members in athletes' support system is important for successful athlete-support. Arguably, sport management is essential to keep athletes informed and at the same time work to establish resources needed at the organizational level. The COVID-19 situation also revealed that decisions of authorities in various countries can greatly impact athletes' preparations and performance at the Olympic Games. For example, the central health authorities in Norway trumped decisions made by local health authorities and the Minister of Health trumped the Minister of Culture that sport usually rely upon. The changed power relationship resulted in constantly changing regulations, causing frustration among athletes and federations (Kristiansen and Lundqvist, 2022).

This study uncovers experiences reported in interviews with 12 Scandinavian elite athletes and the results display naturalistic generalizability to situations and samples that resemblances the ones presented (Smith, 2018). Mental health expressions and needs may, however, be dependent on cultural contexts whereby future studies are encouraged to investigate athletes from various nationalities or elite sports systems. The pandemic undeniably exposed athletes and everyone involved to several psychological strains. The pandemic also provided lessons on how highly stressful and uncertain situations can be managed and enabled the development of novel and innovative initiatives to support to athletes in this extraordinary period in sports history. The holistic perspectives obtained in this study, and the similar experiences expressed from both Norwegian and Swedish athletes, contribute to an increased understanding of elite sport athletes' needs and challenges in stressful and unforeseen situations such as a pandemic. The findings can therefore inform researchers, practitioners, and stakeholders in the development of interventions and support programs for elite athletes' mental health and performance.

## Data Availability Statement

Due to the nature of this research and ethical reasons when the data includes information that could reveal participants identities, sharing of data publicly is not possible. Requests to access the datasets should be directed to carolina.lundqvist@liu.se.

## Ethics Statement

The studies involving human participants were reviewed and approved by the Ethics Review Authority in Sweden and Norway. The patients/participants provided their informed consent to participate in this study.

## Author Contributions

CL and EK both planned the study, recruited participants, performed interviews, conducted data analysis, and reviewed and revised the manuscript multiple times before submission. CL wrote the initial draft with assistance from EK. All authors approved the final manuscript for submission.

## Conflict of Interest

The authors declare that the research was conducted in the absence of any commercial or financial relationships that could be construed as a potential conflict of interest.

## Publisher's Note

All claims expressed in this article are solely those of the authors and do not necessarily represent those of their affiliated organizations, or those of the publisher, the editors and the reviewers. Any product that may be evaluated in this article, or claim that may be made by its manufacturer, is not guaranteed or endorsed by the publisher.

## Footnotes

^1^Initially, the athlete needed to go into a 10-days ‘at home' quarantine after travels abroad. Then this was softened by the athletes being able to test negative and leave quarantine after seven then four days – so the athletes could go back to training. ‘At home' quarantine meant that they could only travel between their homes and training facilities.
